# Overweight, obesity, and colorectal cancer screening: Disparity between men and women

**DOI:** 10.1186/1471-2458-4-53

**Published:** 2004-11-08

**Authors:** Moonseong Heo, David B Allison, Kevin R Fontaine

**Affiliations:** 1 Department of Psychiatry, Weill Medical College of Cornell University, White Plains, NY, USA; 2 Department of Biostatistics, Section on Statistical Genetics & Clinical Nutrition Research Center, University of Alabama at Birmingham, Birmingham, AL, USA; 3 Division of Rheumatology, Johns Hopkins University School of Medicine, Baltimore, MD, USA

## Abstract

**Background:**

To estimate the association between body-mass index (BMI: kg/m^2^) and colorectal cancer (CRC) screening among US adults aged ≥ 50 years.

**Methods:**

Population-based data from the 2001 Behavioral Risk Factor Surveillance Survey. Adults (N = 84,284) aged ≥ 50 years were classified by BMI as normal weight (18.5–<25), overweight (25–<30), obesity class I (30–<35), obesity class II (35–<40), and obesity class III (≥ 40). Interval since most recent screening fecal occult blood test (FOBT): (0 = >1 year since last screening vs. 1 = screened within the past year), and screening sigmoidoscopy (SIG): (0 = > 5 years since last screening vs. 1 = within the past 5 years) were the outcomes.

**Results:**

Results differed between men and women. After adjusting for age, health insurance, race, and smoking, we found that, compared to normal weight men, men in the overweight (odds ratio [OR] 1.25, 95% CI = 1.05–1.51) and obesity class I (OR = 1.21, 95% CI = 1.03–1.75) categories were more likely to have obtained a screening SIG within the previous 5 years, while women in the obesity class I (OR = 0.86, 95%CI = 0.78–0.94) and II (OR = 0.88, 95%CI = 0.79–0.99) categories were less likely to have obtained a screening SIG compared to normal weight women. BMI was not associated with FOBT.

**Conclusion:**

Weight may be a correlate of CRC screening behavior but in a different way between men and women.

## Background

Colorectal cancer (CRC) is the third most common cancer in the United States with approximately 150,000 cases annually leading to about 57,000 annual deaths [1]. A prospective study of over 900,000 US adults found that, compared to normal weight adults, death rates from CRC were 20% to 84% higher in overweight and severely obese men and 10% to 46% higher in overweight and severely obese women [2]. Although other factors (e.g., age, family history) also contribute to CRC risk, obesity is a significant risk factor [1]. Thus, overweight and obese adults should consider obtaining regular CRC screening because early detection and intervention might reduce mortality [1,3].

However, studies suggest that overweight and obese women are more likely to delay cervical and breast cancer screenings than normal weight women [4,5]. In contrast, data from the 2001 Behavioral Risk Factor Surveillance Survey (BRFSS) indicates that, among men, overweight and obesity associates with obtaining prostate-specific antigen testing (Fontaine, Heo & Allison, under review). Although these cancers are sex-specific, the disparity led us to evaluate whether the obesity CRC screening association differed between men and women.

## Methods

The Center for Disease Control and Prevention's BRFSS collects state-based data on preventive health practices and risk behaviors in the non-institutionalized civilian population aged ≥ 18 years [6]. The analyses we report are derived from the 2001 BRFSS. Information on BRFSS design and sampling methods are reported elsewhere [7,8].

### Study variables

Body mass index (BMI; kg/m^2^), calculated from self-reported weight and height, was the predictor.

Outcomes were interval since the most recent use of fecal occult blood test (FOBT), and sigmoidoscopy (SIG) in adults aged ≥ 50 years who reported ever having had the respective screening examination. BRFSS codes FOBT responses as: 'within past year', 'within past 2 years', 'within past 5 years', '5 or more years ago', 'don't know/not sure', or 'refused'. SIG is coded as: 'within past year', 'within past 2 years', 'within past 5 years', 'within past 10 years', '10 or more years ago', 'don't know/not sure', or 'refused'. Consistent with screening recommendations [1], we dichotomized FOBT as 0 = > 1 year since last screening vs. 1 = screened within the past year. For SIG, the American Cancer Society recommends screening every 5 years for adults aged ≥ 50^1^. Thus, SIG was dichotomized as 0 = > 5 years since last screening vs. 1 = screened within the past 5 years.

We included age, education, race, income, self-reported general health status, smoking, employment, and health insurance as covariates.

### Statistical analysis

We grouped respondents into 5 BMI-defined categories (18.5–<25 "normal weight", 25–<30 "overweight", 30–<35 "obesity class I", 35–<40 "obesity class II", and ≥ 40 "obesity class III"). Respondents (n = 250; .3%) with BMI's <18.5 ("underweight") were omitted from the analyses.

We used multivariate logistic regression to estimate BMI-screening associations by entering the BMI-defined categories and potential confounders into the model as either continuous (e.g., age [including polynomials up to the third order]) or dichotomous variables (e.g., health insurance). Using the guidelines proposed by Greenland [9], we retained covariates that were statistically significant at the two-sided 0.20 alpha level or caused a ≥ 10% change in any of the BMI-defined categories when deleted. As a result, education, income, self-reported general health, and employment were omitted. Responses coded as 'don't know/not sure', or 'refused' were treated as missing variables and excluded from analyses, as were respondents with missing data on any covariates. To ensure unbiased general population estimates, we used sample weights provided by the BRFSS. BMI categories were investigated as 4 contrasts with the normal weight category serving as the referent.

To evaluate whether sex moderated the BMI-screening association, we ran adjusted logistic models that also included BMI × sex interaction terms. Finally, because we observed a significant BMI × sex interaction, we then analyzed the data for men and women separately. Analyses were performed with SPSS 11.5.

## Results

The mean age of the respondents was 65 years (median = 63). The mean BMI was 30.2 (median = 31) and 93% reported having health insurance. Less than half reported ever having either a screening FOBT or SIG (Table 1). Among those who ever had a screening examination 54.1% of men and 52.7% of women (χ^2^(1) = 6.61, p = .010) reported obtaining a screening FOBT within the previous year, and 84.4% of men and 80.3% of women (χ^2^(1) = 98.4, p < 0.001) reported obtaining a screening SIG within the previous 5 years.

**Table 1:** Selected characteristics of respondents aged ≥ 50 years

**Characteristic**	Value*	**N**
Age, yrs	64.6 ± 10.1	84,284
Body mass index (BMI), kg/m^2^	30.2 ± 6.2	84,284
Sex, %		
Men	38.2	32,179
Women	61.8	52,106
Race, %		
White	82.3	68,639
Non-white	17.7	14,778
Health insurance, %		
Yes	93.0	78,260
No	7.0	5,904
Smoking, %		
Current smoker	18.2	15,265
Former smoker	35.4	29,709
Never smoker	46.4	38,959
Ever had fecal occult blood test (FOBT), %		
Yes	43.0	37,498
No	53.9	49,123
Ever had screening sigmoidoscopy (SIG), %		
Yes	45.3	39,574
No	51.1	46,584
Screening fecal occult blood test (FOBT), %		
within past year	53.2	18,449
greater that 1 year	46.8	16,238
Screening sigmoidoscopy (SIG), %		
within past 5 years	81.8	30,465
greater than 5 years	18.2	6,771

* Plus-minus values are means ± standard deviation

BMI was not associated with obtaining a FOBT (OR's ranged from 0.90 to 0.98). Compared to normal weight adults, however, those in the overweight (OR = 1.15, 95%CI 1.02–1.31), obesity class I (1.21, 95%CI 1.09–1.35), II (1.17, 95%CI 1.04–1.44) and III (1.27, 95%CI 1.05–1.58) categories were more likely to have obtained a screening SIG within the previous 5 years (p's < 0.05).

**Figure 1:**
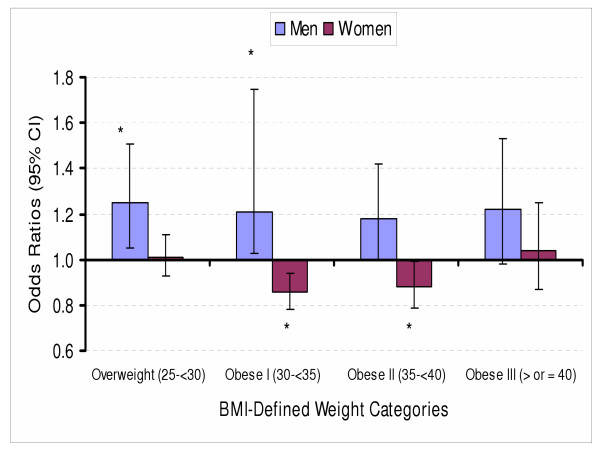
Adjusted odds ratios (OR) for obtaining a screening sigmoidoscopy according to BMI-defined categories for men and women * Significantly different from normal weight reference group at p < 0.05.

The interaction effect between sex and BMI categories on FOBT was not significant (χ^2^(4) = 8.64, p=.071). However, the interaction effect between sex and BMI categories on SIG screening was significant, (χ^2^(4) = 114.03, p < .0001). BMI was not associated with obtaining a FOBT for either sex (OR's ranged from 0.87 to 1.05). However, compared to normal weight men, men in the overweight (1.25, 95%CI 1.05–1.51) and obesity class I (1.21 95%CI 1.03–1.75) categories were significantly more likely to have obtained a screening SIG. In contrast, obesity class I (0.86 95%CI 0.78–0.94) and II (0.88 95%CI 0.79–0.99) women were less likely to have obtained a screening SIG compared to normal weight women (see Figure 1).

## Discussion

These data support an association between BMI and obtaining a screening SIG within the previous 5 years, after smoking, health insurance, race, and age are taken into account. Moreover, the BMI-SIG associations were different between women and men. Women in the obesity class I and II categories were less likely to obtain SIG screening as a function of BMI. This is consistent with associations between BMI and delayed cervical and breast cancer screening [4,5]. On the other hand, men in the overweight and obesity class I categories were more likely to obtain a screening SIG.

The reasons for this disparity are unclear. Perhaps physicians encourage cancer screening more vigorously among their overweight and obese male patients. Differences between men and women on factors such as self-esteem and body image [10] may also contribute to explaining the differential BMI-screening associations. These speculations underscore the importance of identifying barriers that might deter overweight and obese women from obtaining screenings.

This study has limitations including: the BRFSS, a telephone survey, is prone to measurement error; because the BRFSS is an observational study, the BMI-screening associations could be due to residual confounding or confounding from unmeasured variables; the cross-sectional design did not allow testing causal inferences; and people without telephones, approximately 3% of the US population [6], are not surveyed through BRFSS.

## Conclusions

These data indicate that weight may be a correlate of CRC screening behavior but in a different way for men and women.

## Competing interests

The author(s) declare that they have no competing interests.

## Authors' contributions

MH drafted the paper and assisted with the statistical analysis and interpretation. DB assisted with the writing of the manuscript and in the interpretation of the results. KF obtained and analyzed the data and assisted with the preparation of the manuscript.

## Pre-publication history

The pre-publication history for this paper can be accessed here:


